# Evaluating Drug Interactions between Ritonavir and Opioid Analgesics: Implications from Physiologically Based Pharmacokinetic Simulation

**DOI:** 10.3390/ph17050640

**Published:** 2024-05-15

**Authors:** Liang Ni, Zhihai Cao, Jiakang Jiang, Wei Zhang, Wei Hu, Qian Zhang, Chaozhuang Shen, Xijing Chen, Liang Zheng

**Affiliations:** 1 Clinical Pharmacokinetics Laboratory, School of Basic Medicine and Clinical Pharmacy, China Pharmaceutical University, Nanjing 210009, China; 3120090289@stu.cpu.edu.cn; 2 Department of Clinical Pharmacology, The Second Affiliated Hospital of Anhui Medical University, Hefei 230601, China; caozh0826@gmail.com (Z.C.); mathdrug02@gmail.com (W.Z.); huwei@ahmu.edu.cn (W.H.); zhangqian@ahmu.edu.cn (Q.Z.); 3 School of Pharmacy, Anhui Medical University, Hefei 230032, China; 4 Department of Pharmacy and Biomedical Engineering, Clinical College of Anhui Medical University, Hefei 230031, China; jiakangjiang2@gmail.com; 5 Department of Clinical Pharmacy and Pharmacy Administration, West China School of Pharmacy, Sichuan University, Chengdu 610041, China; 2023324050039@stu.scu.edu.cn

**Keywords:** drug–drug interactions, ritonavir, fentanyl, hydrocodone, PBPK, modeling and simulation

## Abstract

Several commonly used opioid analgesics, such as fentanyl, sufentanil, alfentanil, and hydrocodone, are by report primarily metabolized by the CYP3A4 enzyme. The concurrent use of ritonavir, a potent CYP3A4 inhibitor, can lead to significant drug interactions. Using physiologically based pharmacokinetic (PBPK) modeling and simulation, this study examines the effects of different dosing regimens of ritonavir on the pharmacokinetics of these opioids. The findings reveal that co-administration of ritonavir significantly increases the exposure of fentanyl analogs, with over a 10-fold increase in the exposure of alfentanil and sufentanil when given with ritonavir. Conversely, the effect of ritonavir on fentanyl exposure is modest, likely due to additional metabolism pathways. Additionally, the study demonstrates that the steady-state exposure of hydrocodone and its active metabolite hydromorphone can be increased by up to 87% and 95%, respectively, with concurrent use of ritonavir. The extended-release formulation of hydrocodone is particularly affected. These insights from PBPK modeling provide valuable guidance for optimizing opioid dosing and minimizing the risk of toxicity when used in combination with ritonavir-containing prescriptions.

## 1. Introduction

Opioids are a class of drugs that are widely used for the management of moderate to severe pain. These medications act on the central nervous system to reduce the perception of pain and include both natural and synthetic compounds. Fentanyl, sufentanil, alfentanil, and hydrocodone are examples of commonly used opioid analgesics that are by report primarily metabolized by the cytochrome P450 3A4 (CYP3A4) enzyme [1,2]. Protease inhibitors have become a key component of highly active antiretroviral therapy (HAART) for the long-term treatment of AIDS. Ritonavir is an HIV-1 protease inhibitor that is primarily used in combination with other antiretroviral drugs to treat HIV infection. Notably, it is commonly used as a booster to increase the plasma concentrations of other protease inhibitors. This effect is achieved through its strong inhibition on CYP3A4. Therefore, coadministration with ritonavir can lead to increased plasma concentrations of some opioids, such as fentanyl and oxycodone, by lowering the CYP3A4-mediated clearance of these drugs [3,4]. In reality, concurrent use of ritonavir or ritonavir-containing prescriptions and opioid analgesics is prevalent in substance abusers and HIV-infected individuals who require pain management [5,6,7]. On the other hand, a real-world analysis discovered that the top 100 medications provided to US COVID-19 patients who received concurrent nirmatrelvir/ritonavir therapy include many opioids, such as oxycodone, hydrocodone, tramadol, codeine, and fentanyl [8]. This reflects the actual prevalence of opioid analgesic use. Sometimes, there will be no turning back the necessity for medical intervention. Therefore, clinicians should be aware of any possible interactions between ritonavir and anesthesia-related medications and modify perioperative treatment accordingly.

The therapeutic efficacy and side effects of opioids are closely related to their systemic exposure [9]. Common adverse events related to opioids include respiratory depression, oversedation, nausea, vomiting, constipation, and urinary retention. In severe cases, these can lead to life-threatening complications such as respiratory arrest and cardiovascular events. Additionally, long-term use of opioids can lead to physical dependence, tolerance, and the potential for addiction [10]. Adverse events related to ritonavir alone include gastrointestinal disturbances such as nausea, diarrhea, and abdominal pain. It can also cause metabolic changes, including hyperlipidemia and insulin resistance, which may increase the risk of cardiovascular disease [11]. The problematic aspect of the interaction between opioids and ritonavir mainly arises from the inhibition of CYP3A4 by ritonavir. The increased exposure to opioids may significantly enhance the risk of adverse events, particularly respiratory depression and oversedation, which can be life-threatening [9,12]. Moreover, the interaction can lead to undiscovered pharmacokinetics, making it challenging to achieve the desired therapeutic effect while minimizing the risk of adverse events.

Fentanyl, sufentanil, and alfentanil are all synthetic opioids with a potency many times greater than morphine [13]. Healthcare professionals typically administer them in a controlled setting due to the risks involved, including respiratory depression, which can be life-threatening. Fentanyl is thought to be primarily metabolized by CYP3A4, while sufentanil and alfentanil are eliminated exclusively by CYP3A4 [14,15,16]. The medication inserts for these drugs all indicate a risk of DDI in combination with CYP3A4 perpetrators. Hydrocodone is a semi-synthetic opioid derived from codeine, which is itself a naturally occurring substance extracted from the poppy plant [17]. Both hydrocodone and its active metabolite, hydromorphone, are effective μ-opioid receptor agonists. Hydrocodone can be taken orally as tablets, capsules, or liquid solutions. It is also available in combination with non-opioid analgesics such as acetaminophen (paracetamol) or ibuprofen. Hydrocodone is metabolized primarily in the liver by the cytochrome P450 enzyme system, specifically the CYP3A4 isoenzyme, to norhydrocodone. Hydromorphone is formed via CYP2D6, which only contributes to around 3% of the total clearance of hydrocodone [18]. Hydrocodone also has a non-CYP hepatic clearance that has not been fully clarified [19]. Hydromorphone is a more potent analgesic than morphine and hydrocodone, with a higher oral potency and a faster onset of action. It is metabolized in the liver primarily by glucuronyl transferase (UGT) 2B7 to form inactive 3-glucuronide [20]. This conversion is mediated by glucuronosyltransferase and is less likely to be affected by drug interactions. Previous studies indicated that multiple doses of ritonavir increase single-dose fentanyl and hydrocodone plasma exposures by 83% and 90%, respectively [3,21]. Although the effect of co-administering ritonavir on sufentanil and alfentanil is unknown, it is anticipated to be more substantial. 

Physiologically based pharmacokinetic (PBPK) modeling and simulation is an advanced pharmacological tool that integrates various physiological, anatomical, and biochemical data to predict the fate of drugs in the body [22]. PBPK models have been increasingly utilized to evaluate drug interactions, providing a mechanistic understanding of the underlying processes and enabling the prediction of drug concentrations under different scenarios [23]. This approach can promisingly assist healthcare professionals in making informed decisions regarding drug dosing, scheduling, and selection [24]. For instance, we previously created and validated a ritonavir PBPK model incorporating CYP3A4 and CYP2D6 modulation and used it to predict ritonavir–oxycodone interactions [25]. The model closely predicts the exposure change of oxycodone in the presence of ritonavir, as seen in clinical investigations. The model unraveled hitherto unstudied exposure-related dangers of oxycodone–ritonavir interactions and directed appropriate concurrent dosage.

The objective of this study is to evaluate the drug interactions between ritonavir and commonly used opioid analgesics, such as fentanyl, sufentanil, alfentanil, and hydrocodone, using PBPK modeling and simulation. Specifically, we aimed to assess the impact of different dosing regimens of ritonavir on the pharmacokinetics of these opioids, with a focus on understanding the potential changes in opioid exposure, which may be beneficial information for clinical practice where ritonavir-containing prescriptions are used.

## 2. Results

### 2.1. Hydrocodone and Hydromorphone Model Development and Evaluation

**Figure 1:**
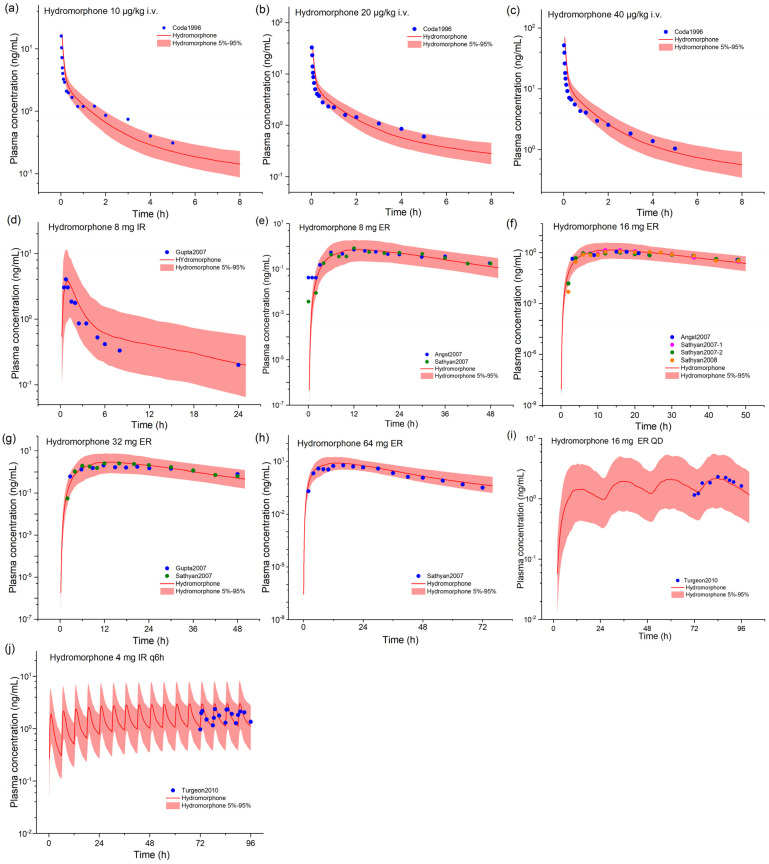
The observed and model-predicted plasma concentration–time profiles of hydromorphone. The dark lines indicate median concentration data from model predictions, while the shaded area indicates a 5% to 95% concentration range. The dots represent previously reported concentration data, and the source literature is provided in the Supplemental Materials. IR, immediate release; ER, extended release. (**a**–**j**) depict simulations for various scenarios, with dosage information supplied in the Supplementary Materials.

By combing reported and optimized compound-specific parameters, we developed hydromorphone and hydrocodone PBPK models in the PK-Sim^®^. Figure 1 shows the blood concentration–time profiles of hydromorphone intravenous injection and oral administration of single- or multi-dose immediate- and extended-release formulations simulated by the established hydromorphone PBPK model. Compared to the observed data, the model simulation results are acceptable and can replicate the results of various oral dose regimens for hydromorphone. The predicted and observed values of the main pharmacokinetic parameters are listed in Supplementary Table S1. The fold errors (FEs) of the predicted/measured pharmacokinetic parameters maximum concentration (C_max_), area under the curve (AUC), and peak time (T_max_) for the hydromorphone model are in the range of 0.76–1.28, 0.85–1.29, and 0.90–1.23, respectively. The geometric mean fold errors (GMFEs) of C_max_, AUC, and T_max_ for all the simulation studies are 1.17, 1.08, and 1.09, respectively, indicating good model predictive performance. According to the sensitivity analysis (Figure S1), hydromorphone exposure is sensitive to UGT2B7-mediated clearance, lipophilicity, plasma unbound fraction, and specific intestinal permeability, apart from formulation-related parameters.

**Figure 2:**
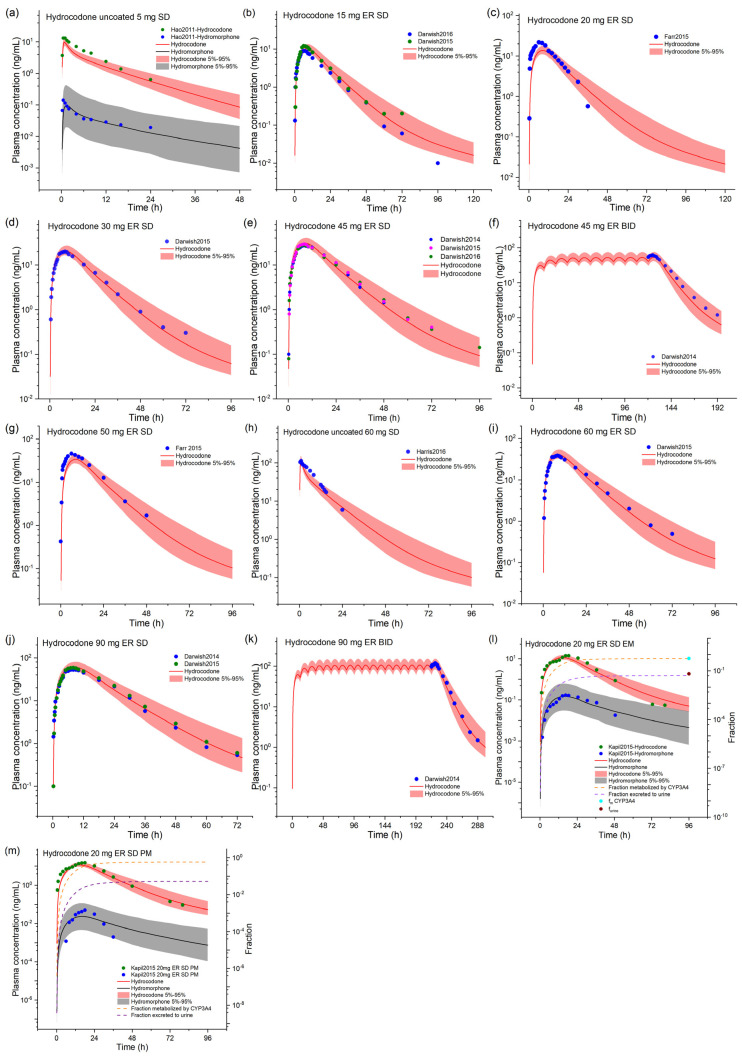
The observed and model-predicted plasma concentration–time profiles of hydrocodone and its metabolite hydromorphone. The dark lines indicate median concentration data from model predictions, while the shaded area indicates a 5% to 95% concentration range. The dots represent previously reported concentration data, and the source literature is provided in the Supplemental Materials. ER, extended release; f_m,CYP3A4_, fraction metabolized by CYP3A4; f_urine_, fraction excreted via urine; SD, single dose. (**a**–**m**) depict simulations for various scenarios, with dosage information supplied in the Supplementary Material.

For hydrocodone, we observed that the model achieved good simulation of observed blood concentrations under various dosing scenarios (Figure 2). The pharmacokinetic parameters are shown in Table S2. The FEs of C_max_, AUC, and T_max_ for the predicted/measured values of the hydrocodone model are in the range of 0.60–1.10, 0.69–1.24, and 0.50–1.83, respectively, and the GMFEs of C_max_, AUC, and T_max_ for all the simulation studies are 1.18, 1.16, and 1.23, respectively. We can visualize a significant decrease in the hydromorphone concentration after administering hydrocodone in CYP2D6 poor metabolizers (PM, proximity to co-administration with paroxetine). The model predicts that hydromorphone AUC after a single oral dose of hydrocodone in CYP2D6 extensive metabolizers (EM) is 5.8-fold higher than in PM. CYP2D6 PM has no significant impact on the fraction metabolized by CYP3A4 and excreted via the kidneys, consistent with the previous reports. The GMFEs of C_max_, AUC, and T_max_ for the metabolite hydromorphone are 1.79, 1.22, and 1.21, respectively. The GMFEs range from 0.5 to 2, indicating the model’s good predictability. Hydrocodone AUC is most sensitive to the pKa value due to the cellular permeability calculation method selected, followed by dose, plasma unbound fraction, CYP3A4 metabolism kinetics, and lipophilicity (Figure S1). 

### 2.2. Model-Based DDI Prediction between Ritonavir and Fentanyl Analogs

We used PBPK modeling to simulate DDI between ritonavir and fentanyl/alfentanil/sufentanil. First, We tested the impact of ritonavir on fentanyl pharmacokinetics using a clinical study with the following dosing regimen: Ritonavir administered for three consecutive days, 200 mg t.i.d. of ritonavir on day 1, 300 mg t.i.d. on day 2, and 300 mg as a single dose on day 3, and fentanyl was administered i.v. 5 μg/kg 2 h after the second dose of ritonavir on day 2 [3]. As shown in Figure 2, the predicted blood concentrations in the control group closely matched the measured values from the pharmacokinetic study. However, the predicted blood concentrations in the ritonavir group were lower than the measured values from the same study, leading to the underestimation of the AUC ratio (AUCR) of fentanyl (Table 1). 

**Figure 3:**
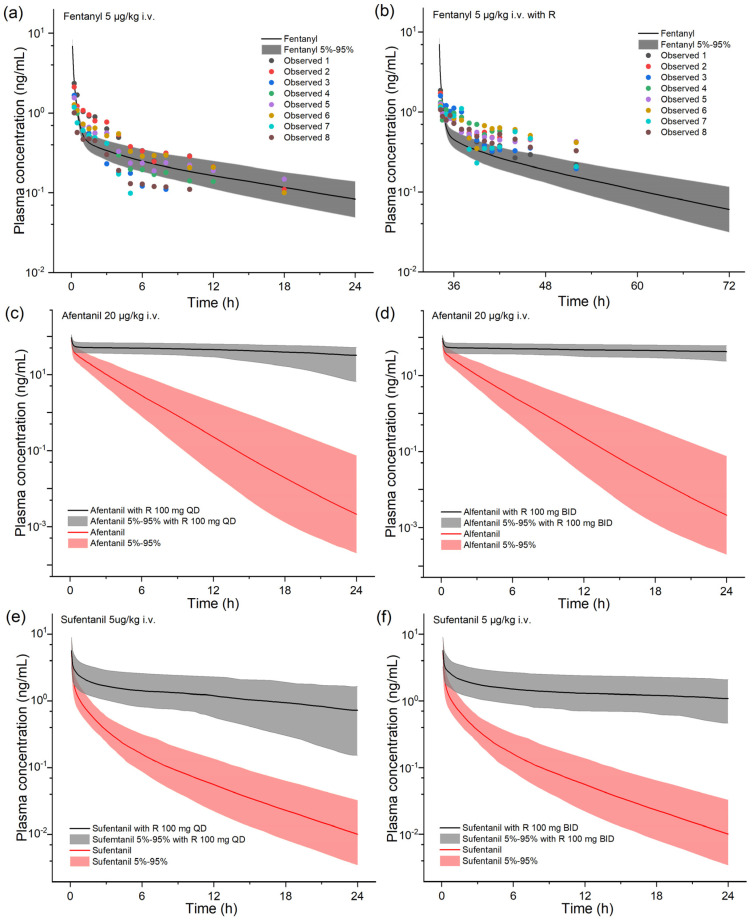
Model-predicted plasma concentration–time profiles of selected fentanyl analgesics following single administration in the presence of ritonavir (100 mg QD or BID). Population simulation results are presented as the median with a 5~95% concentration range. R, ritonavir.

We simulated the exposure change of alfentanil/sufentanil when combined with a standard dosing regimen of ritonavir 100 mg BID or QD. In this simulation, alfentanil/sufentanil was given after three doses of ritonavir when the inhibition of CYP3A4 reached a stable maximum level. The results are shown in Figure 3 and Table 2. The co-administration of sufentanil/alfentanil and ritonavir led to a remarkable increase in overall exposure (10- to over 50-fold) and half-life in opioids compared to sufentanil/alfentanil monotherapy. The ability to maintain relatively high blood concentrations and a slower decline in drug concentration suggests that in vivo clearance of sufentanil/alfentanil is significantly inhibited during continuous ritonavir administration. As ritonavir is a time-dependent inhibitor of CYP3A4, we also modeled the duration after ritonavir withdrawal, during which these fentanyl analogs could be used as usual. For sufentanil as an example, 84 h after the last dose of the ritonavir QD regimen and 96 h after the last dose of the ritonavir BID regimen, followed by sufentanil injections, did not result in a statistically significant change in sufentanil exposure in the population (less than 25%).

### 2.3. Model-Based DDI Prediction between Ritonavir and Hydrocodone

Our established PBPK model simulated the effect of ritonavir co-medication on hydrocodone pharmacokinetics. First, a validation simulation was conducted using the following protocol: Ritonavir 100 mg daily was given for 16 consecutive days, and hydrocodone uncoated tablet on day 15 co-administered with ritonavir [21]. We observe a modest increase in hydrocodone plasma concentrations in the presence of ritonavir, as shown in Figure S2. The simulated C_max_R and AUCR are 1.24 and 1.81, respectively, compared to the reported values of 1.27 and 1.90. 

**Figure 4:**
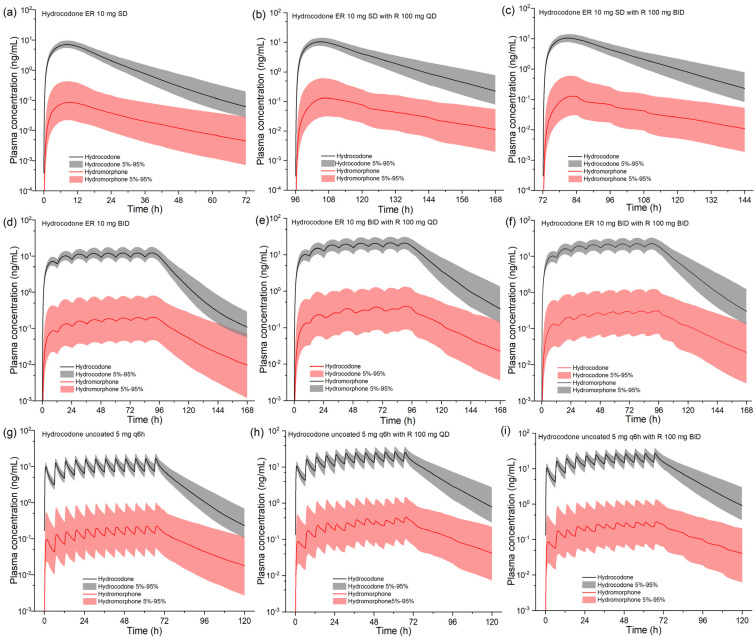
Model-predicted plasma concentration–time profiles of hydrocodone and its metabolite hydromorphone following single and multiple oral administrations of hydrocodone in the presence of ritonavir. Population simulation results are presented as the median with a 5~95% concentration range. R, ritonavir; ER, extended release.

The model was then extrapolated to explore unstudied co-administration scenarios. Figure 4 shows the impact of ritonavir 100 mg QD or BID on the pharmacokinetics of consecutive doses of hydrocodone uncoated or ER tablets. Table 3 lists the pharmacokinetic parameters of hydrocodone and hydromorphone. For the single-dose hydrocodone ER tablet, ritonavir has a more significant effect on hydrocodone C_max_ than the uncoated formulation (46% increase vs. 27% increase). Ritonavir increases the steady-state C_max_ of hydrocodone by 73~81% and 54~60% for ER and uncoated formulation, respectively, indicating more profound effects on multiple doses of hydrocodone. Ritonavir also prolongs the half-life of hydrocodone, especially during its multiple administration. The total exposure (AUC) was increased by nearly 80% under various scenarios. Furthermore, according to the simulation, ritonavir increases hydromorphone exposure to various degrees.

## 3. Discussion

This study utilized PBPK modeling to simulate the effect of ritonavir on the pharmacokinetics of selected opioids whose in vivo clearance is highly dependent on CYP3A4. Clinical trials have not thoroughly investigated these co-medication scenarios. 

We examined the altered biological exposure of fentanyl analgesics in the presence of co-administration of ritonavir, of which no clinical DDI studies have been reported for alfentanil and sufentanil. By using PBPK modeling, we reveal that the significant increase in blood concentrations of these two drugs when co-administered with ritonavir produces a clinically significant risk of cautionary drug–drug interactions. The AUC of alfentanil and sufentanil administered as a single injection can be increased by more than 10-fold under the clinically common 100 mg QD or BID dosing regimen of ritonavir, so their use should be with extreme caution or better avoided when ritonavir-containing protease inhibitors are used in patients. Compared to alfentanil and sufentanil, fentanyl dosing is relatively safer for prescription with ritonavir. The PBPK model underestimated the change in fentanyl exposure in the presence of ritonavir, with a clinically measured AUCR of 2.70 and a model-predicted AUCR of 1.28. Fentanyl is a classic opioid that has been developed and marketed for decades, but its in vivo disposition has not been fully understood. CYP enzymes metabolize fentanyl into at least three metabolites, and the metabolizing enzyme that has been identified is CYP3A4. Although CYP3A4 is generally recognized in the literature as the major metabolizing enzyme for fentanyl, the exact magnitude of CYP3A4’s contribution to the total clearance is unclear. The published PBPK model for fentanyl underwent rigorous modeling evaluation and closely predicted the extent of drug–drug interactions with voriconazole, a moderate inhibitor of CYP3A4 [26]. According to the model prediction, the contribution of CYP3A4 metabolism accounts for less than 30% of overall clearance. This is a significant factor in the modest impact of ritonavir on the levels of fentanyl in the blood that the model predicted. On the other hand, clinical studies have shown that other potent inhibitors of CYP3A4 also have a minimal effect on fentanyl. For example, co-administration of itraconazole had essentially no effect on fentanyl blood concentrations, and co-administration of ketoconazole caused only a 33% increase in fentanyl AUC [27,28]. It implies that the fentanyl in vivo clearance is not highly dependent on CYP3A4 and that other, as-yet-unidentified variables may also play a role in the way ritonavir affects fentanyl pharmacokinetics. When sufentanil and alfentanil are cleared essentially exclusively by CYP3A4, the effect of ritonavir is significant. We also estimated the duration of time these fentanyl analogs may be used routinely following ritonavir withdrawal, as ritonavir is a time-dependent inhibitor of CYP3A4. The results remind clinicians that alfentanil with sufentanil should also be avoided for a considerable period after discontinuing ritonavir-containing preparations.

Hydrocodone is metabolized to norethindrone by CYP3A4-mediated N-demethylation and to hydromorphone by CYP2D6-mediated O-demethylation. When ritonavir and hydrocodone are combined, the clearance of the latter decreases. As a result, smaller doses of hydrocodone combined with a sensible dosing regimen are required to achieve optimal therapeutic efficacy and prevent opioid-related side effects. The model we developed closely predicts changes in exposure to hydrocodone in the presence of ritonavir. Based on the results of model simulations, the steady-state exposures of hydrocodone and hydromorphone can be increased by up to approximately 87% and 95%, respectively, when co-administered with ritonavir. Oral extended-release formulations of hydrocodone produce higher plasma concentrations and lower peak-to-valley variability over the dosing interval than oral uncoated formulations of hydrocodone. Opioid extended-release formulations have the specific advantage of producing more consistent pain relief, improving sleep, and can lead to fewer AEs. In contrast, immediate-release formulations provide rapid and effective pain relief. In addition, hydrocodone immediate-release formulations reach peak plasma concentrations in a short period, have a shorter half-life, and are given repeatedly for short periods for adequate pain control. These pharmacokinetic properties may contribute to the potential for abuse; the rapid onset of action may result in a more rapid pleasurable effect, and the short half-life requiring repeated dosing may promote continued use. On the other hand, hydrocodone is frequently in combination with acetaminophen. Although ritonavir has no obvious effect on acetaminophen pharmacokinetics, the fixed-dose combination makes it less likely to change the hydrocodone dose on its own. As a result, when using ritonavir-containing medicines, acetaminophen/hydrocodone tablets should be avoided to the greatest extent possible.

Since hydromorphone is primarily metabolized through glucuronidation mediated by UGT2B7, we did not consider the effect of ritonavir on hydromorphone metabolism. Though hydrocodone has a hepatic clearance by non-CYP enzyme, and the impact of ritonavir on this part of metabolism is unknown, the PBPK prediction for ritonavir–hydrocodone DDI is precise. Despite ritonavir being a moderate competitive inhibitor of CYP2D6, exposure to the metabolite hydromorphone was instead elevated when ritonavir was co-administered. The reason might be that, following the inactivation of the primary metabolizing enzyme of hydrocodone, CYP3A4, the competitive inhibition of CYP2D6 by ritonavir was insufficient to offset the effect of the elevated concentration of the parent drug as the substrate. CYP2D6 inhibition contributes little to the increase in exposure to hydrocodone, according to the model. Hydromorphone is active, but its in vivo exposure is equivalent to less than 2% of the parent drug. Previous studies also indicated that CYP2D6 poor metabolizers did not affect the efficacy of hydrocodone [29]. Therefore, it is reasonable to adjust the dosing regimen solely based on hydrocodone exposure.

Given the potential for significant drug interactions, prescribers should be aware of the following considerations when managing patients on ritonavir-containing regimens. First, prescribers should consider reducing the dose of opioid analgesics when co-administering with ritonavir, especially for those with a narrow therapeutic index or those known to be highly dependent on CYP3A4 for metabolism. Second, enhanced monitoring for signs of opioid toxicity, such as respiratory depression, oversedation, and other adverse effects, is essential. This may include more frequent clinical assessments and the use of objective measures of respiratory function. In cases where the risk of interaction is deemed too high, prescribers may consider alternative analgesics that are less dependent on CYP3A4 for metabolism, such as remifentanil [30]. Third, patients should be educated about the potential risks of combining these medications and the importance of adhering to the prescribed dosing regimen. The PBPK simulation from this study may provide valuable information for optimizing the clinical use of these medications and ensuring patient safety.

The limitations of this study include the following: (1) More in vitro research is needed to determine the precise cause of the model’s underestimation of ritonavir’s impact on fentanyl blood concentrations and to confirm our deductions. (2) Ritonavir is seldom used alone nowadays. While other protease inhibitors affect CYP enzyme activity to varying degrees, this study does not explore this topic. However, ritonavir alone can strongly inhibit CYP3A4 for drugs primarily metabolized by it, and the effect of other protease inhibitors should be minimal. To better understand and manage these interactions, we propose the following areas for future research. First, conducting clinical trials to validate the findings from our PBPK modeling and to establish evidence-based guidelines for the co-administration of ritonavir and opioid analgesics. Second, further refinement of the PBPK models to include additional factors that may influence the interaction. Finally, analysis of real-world data to assess the frequency and severity of adverse events associated with the co-administration of ritonavir and opioid analgesics. 

## 4. Materials and Methods

### 4.1. PBPK Modeling Platform and Related Software

PBPK modeling was performed using the Open Systems Pharmacology (OSP) Suite version 11.0 (PK-Sim^®^ & Mobi^®^, http://www.open-systems-pharmacology.org/, available as freeware under the GPLv2 license). The software offers a comprehensive modeling framework consisting of 17 distinct compartments, each representing an organ or tissue connected to the venous and arterial blood pools. Virtual populations were created using the PK-Sim^®^ physiology engine. In PK-Sim^®^, the Monte Carlo algorithm was used for parameter identification. WebPlotDigitizer version 4.3 (Ankit Rohatgi, Austin, TX, USA) retrieved the observed numerical plasma concentrations (mean values) using published time–concentration curves from earlier clinical investigations.

### 4.2. Drug Model Preparation

Fentanyl, sufentanil, and alfentanil PBPK models were supplied as OSP templates and already validated in previous studies [26,31]. Anyone may download these models straight from the OSP community on GitHub. Their compound-specific parameters are provided in Table S5. We previously developed and validated a ritonavir PBPK model incorporating competitive and time-dependent inhibition and induction effects of CYP3A4 by ritonavir [25]. We used the developed ritonavir model by adding P-glycoprotein inhibition with a reported inhibition constant of 0.20 μmol/L. In this study, we newly developed hydrocodone and hydromorphone PBPK models for DDI prediction. 

First, we developed a PBPK model for hydromorphone. After the model parameters for hydromorphone were determined, the CYP2D6 metabolism parameters for hydrocodone could be subsequently determined, as hydromorphone is exclusively produced via CYP2D6. The physiochemical parameters, including logP, fraction unbound in plasma, solubility, pKa, and molecular weight, were obtained from the literature or Drugbank database (https://go.drugbank.com/, accessed on 5 November 2023). Partition coefficients and cell permeability parameters were estimated using the PK-Sim Standard method. Hydromorphone is mainly cleared by UGT2B7-mediated glucuronidation in vivo [32]. The specific clearance rate for UGT2B7 metabolism was optimized by fitting the observed concentration data according to the Monte Carlo algorithm. Since there was no reported re-absorption process of hydromorphone, the GFR fraction was set as 1.0. Regarding oral formulations, we defined the uncoated tablet as the solution, and the dissolution profile of the sustained-release tablet was described by a Weibull equation. 

For hydrocodone, partition coefficients and cell permeability parameters were estimated using Schmitt and Charge-dependent Schmitt normalized to PK-Sim methods, respectively [33]. In vivo clearance pathways of hydrocodone include CYP3A4, CYP2D6, non-CYP enzyme-mediated metabolism, and renal clearance of the parent drug. The contribution of in vivo clearance outside the CYP pathway is estimated to be at least 40% based on in vitro metabolism data [19]. Based on the proportion of metabolites generated, CYP2D6 (normal metabolic activity) clearance accounts for approximately 3%, from which the contribution of CYP3A4 clearance is calculated to be approximately 57%. Renal clearance of the parent drug accounts for 6.5% [18]. These proportions were used in hydrocodone modeling with test data to aid the optimization of relevant parameters, including metabolic turnover number (kcat) for each metabolic enzyme and clearance for non-CYP metabolism. The kinetics of the CYP3A4 and CYP2D6 enzyme reactions belong to the Michaelis–Menten kinetics, and the Km was obtained from the literature. CYP2D6 is genetically polymorphic, and we set up extensive metabolizer (EM) and poor metabolizer (PM), whose kcat was optimized based on clinically reported blood concentrations of hydromorphone after hydrocodone administration. In the present study, we approximated the blood concentration of hydromorphone in the presence of paroxetine, a CYP2D6 potent inhibitor, as equivalent to that in CYP2D6 PM [34]. Other parameters remained unchanged when CYP2D6 kcat was adjusted to reflect CYP2D6 phenotypes. The GFR fraction was set to the default value (1.0) to reflect the actual fraction of renal clearance for the parent drug. We also optimized the transcellular intestinal permeability of hydromorphone and formulation parameters, i.e., dissolution time and shape of the Weibull equation. The final compound-specific parameters for hydrocodone and hydromorphone are listed in Table 4.

PBPK simulations were conducted using a virtual American population containing 200 individuals aged 18 to 60 to test the model’s predictive performance. The observed data references and the dosing regimen and formulation type are listed in Tables S3 and S4. The fold and geometric fold errors for pharmacokinetic parameters were calculated according to previously described methods [35]. 

**Table 4:** Input compound parameters for the hydromorphone and hydrocodone PBPK models and inhibition/induction kinetics of ritonavir.

Parameters	Hydromorphone	Reference/Source	Hydrocodone	Reference/Source
Lipophilicity	1.8	[36]	2.0	[37]
Plasma fraction unbound	0.86	Drugbank	0.64	Drug label
MW	285.30 g/mol	Drugbank	299.40 g/mol	Drugbank
pKa	8.5	PubChem	8.23	PubChem
Solubility	0.149 mg/mL	PubChem	0.797 mg/mL	PubChem
Specific intestinal permeability	3.27 × 10^−6^ cm/min	Optimized	2.00 × 10^−4^ cm/min	Optimized
Partition coefficients calculation	Diverse	PK-Sim standard	Diverse	Schmitt
Cellular permeability	3.37 × 10^−3^ cm/min	PK-Sim standard	4.00 × 10^−3^ cm/min	Charge-dependent Schmitt normalized to PK-Sim
UGT2B7 specific clearance	6.11 1/min	Optimized	NA	
Formulation	IR dissolved, ER Weibull		Weibull	
50% dissolution time	ER 8.48 h	Optimized	Uncoated 0.80 h, ER 6.40 h	Optimized
Dissolution shape	ER 2.94	Optimized	Uncoated 0.79 ER 1.79	Optimized
GFR fraction	1.0	Assumed	1.0	Assumed
K_m,CYP3A4_	NA		2.60 mmol/L	[19]
K_cat,CYP3A4_	NA		361 1/min	Optimized
K_m,CYP2D6_	NA		54 μmol/L	[19]
K_cat,CYP2D6_ (EM)	NA		7.12 1/min	Optimized
K_cat,CYP2D6_ (PM)	NA		1.07 1/min	Optimized
UGT2B7-specific clearance	6.11 1/min	Optimized	NA	
Hepatic clearance (non-CYP)	NA		0.36 1/min	Optimized
	Ritonavir			
K_i,CYP3A4_	0.25 μM	[38]		
K_inact,CYP3A4_	0.40 1/min	[39]		
K_inact_half,CYP3A4_	0.20 μM	[39]		
K_i,CYP2D6_	0.04 μM	[40]		
EC50_CYP3A4_	0.17 μM	[25]		
Emax_CYP3A4_	7.47	[25]		
K_i,P-gp_	0.20 μM	[41]		

MW, molecular weight; pKa, acid dissociation constant; GFR, glomerular filtration; K_m_, Michaelis–Menten constant; k_cat_, V_max_ per recombinant enzyme; EM, extensive metabolizer; PM, poor metabolizer; IR, immediate release; ER, extended release; K_i_, competitive inhibition constant; K_inact_, maximum inactivation rate constant; K_inact_half_, inactivator concentration yielding half K_inact_; Emax, maximal induction effect; EC50, concentration to reach half Emax; NA, not applicable.

### 4.3. PBPK Modeling-Based Simulation for Ritonavir–Opioid DDIs

We performed population PBPK simulations based on a virtual Caucasian population containing 200 individuals to predict the effects of multiple doses of ritonavir on the pharmacokinetics of selected opioids. We first tested the clinically reported dosing scenarios for ritonavir–fentanyl and ritonavir–hydrocodone interactions. The C_max_ ratio (if applicable) and AUC ratio were calculated as the ratio of the values under co-medication divided by the corresponding control. We then simulated hitherto unstudied co-administration scenarios. Ritonavir quickly achieved the most potent inhibition of CYP3A4 following two doses. One hour after the third dose of ritonavir (QD for 7 days or BID for a total of 10 doses), alfentanil/sufentanil was administered to explore the most significant impact. For hydrocodone, a single-dose hydrocodone ER tablet was given on day 4 a.m. concurrently with ritonavir when the latter was administered QD for 7 days or BID for a total of 10 doses. To examine the steady-state exposure changes of hydrocodone and hydromorphone, multiple doses of hydrocodone were simulated as uncoated tablets q6h (5 mg) or ER tablets BID (10 mg) combined with ritonavir 100 mg QD or BID. The predicted pharmacokinetic parameters were directly obtained from PK-Sim^®^ outputs. 

## 5. Conclusions

The PBPK models have been successfully developed to predict drug interactions between ritonavir and fentanyl analogs/hydrocodone. The model emphasizes the significant risk of administering sufentanil/alfentanil in conjunction with ritonavir. When co-medication is inevitable, the model may have a wide range of implications for optimizing and predicting fentanyl and hydrocodone dosage, while the effect on fentanyl pharmacokinetics needs further research.

## Figures and Tables

**Table 1:** Predicted and observed C_max_R and AUCR of fentanyl and hydrocodone in the presence of ritonavir. NA, not available.

Drugs	C_max_R		AUCR	
	Predicted	Observed	Predicted	Observed
Fentanyl	NA	NA	1.28	2.70
Hydrocodone	1.24	1.27	1.81	1.90

**Table 2:** Model-simulated pharmacokinetic parameters of alfentanil and sufentanil under various dosing scenarios.

Drugs	Protocols	AUC_0~∞_ (ng × h/mL)	T_1/2_ (h)
Alfentanil	Alfentanil 20 μg/kg i.v.	94.9	2.29
	Alfentanil 20 μg/kg i.v. + R 100 mg QD	2717 (+2766%)	17.4 (+660%)
	Alfentanil 20 μg/kg i.v. + R 100 mg BID	5581 (+5787%)	63.7 (+2681%)
Sufentanil	Sufentanil 5 μg/kg i.v.	4.58	5.42
	Sufentanil 5 μg/kg i.v. + R 100 mg QD	59.1 (+1192%)	11.4 (+110%)
	Sufentanil 5 μg/kg i.v. 84 h after R 100 mg QD withdrawal	5.54	5.38
	Sufentanil 5 μg/kg i.v. + R 100 mg BID	117 (+2448%)	39.8 (+634%)
	Sufentanil 5 μg/kg i.v. 96 h after R 100 mg BID withdrawal	5.57	6.46

R, ritonavir. The relative change (%) as compared to alfentanil or sufentanil alone is shown in brackets.

**Table 3:** Model-simulated pharmacokinetic parameters of hydrocodone and hydromorphone under various dosing scenarios. HYD, hydrocodone; HYM, hydromorphone; R, ritonavir.

Protocols	C_max_/C_max-ss_ (ng/mL)		AUC/AUC_ss_ (ng×h/mL)		T_1/2_ (h)	
	HYD	HYM	HYD	HYM	HYD	HYM
HYD ER 10 mg SD	7.11	0.09	135	2.35	13.1	19.7
HYD ER 10 mg SD + R 100 mg QD	10.4 (46.3%)	0.13 (44.4%)	241 (78.5%)	4.22 (79.6%)	12.3 (−6.1%)	18.9 (−4.1%)
HYD ER 10 mg SD + R 100 mg BID	10.5 (46.3%)	0.13 (44.4%)	248 (83.7%)	3.87 (64.7%)	15.7 (19.8%)	20.8 (5.6%)
HYD ER 10 mg BID	12.24	0.20	135	2.24	10.2	13.9
HYD ER 10 mg BID + R 100 mg QD	21.2 (73.2%)	0.39 (95.0%)	235 (74.1%)	3.78 (68.8%)	13.6 (33.3%)	24.1 (73.4%)
HYD ER 10 mg BID + R 100 mg BID	22.1 (80.6%)	0.31 (55.0%)	245 (81.5%)	3.40 (51.8%)	13.6 (33.3%)	24.1 (73.4%)
HYD uncoated 5 mg q6h	17.1	0.23	65.1	1.07	5.73	5.22
HYD uncoated 5 mg q6h + R 100 mg QD	26.4 (54.4%)	0.42 (82.6%)	116 (78.2%)	2.01 (87.9%)	7.11 (24.1%)	8.89 (70.3%)
HYD uncoated 5 mg q6h + R 100 mg BID	27.3 (59.6%)	0.32 (39.1%)	122 (87.4%)	1.60 (49.5%)	7.11 (24.1%)	8.89 (70.3%)

## Data Availability

Hydrocodone and hydromorphone model files will be publicly available at the Open Systems Pharmacology repository on GitHub. Model files for other drugs have been provided elsewhere and described in the article.

## Supplementary Materials

The following supporting information can be downloaded at https://www.mdpi.com/article/10.3390/ph17050640/s1, Figure S1: The sensitivity analysis for PBPK models used in this study; Figure S2: Simulated concentrations of a single-dose hydrocodone uncoated tablet in the presence of ritonavir; Table S1: Predicted and observed values for pharmacokinetic parameters of hydromorphone; Table S2: Predicted and observed values for pharmacokinetic parameters of hydrocodone; Table S3: Clinical pharmacokinetic reports used in hydromorphone PBPK modeling; Table S4: Clinical pharmacokinetic reports used in hydrocodone PBPK modeling; Table S5: Input compound parameters for the fentanyl, alfentanil, and sufentanil PBPK models.
