# Supplementary material for: Evaluating Drug Interactions between Ritonavir and Opioid Analgesics: Implications from Physiologically Based Pharmacokinetic Simulation
Source: Pharmaceuticals (Basel). 2024 May 15;17(5):640. doi: 10.3390/ph17050640 (PMC11124264; doi:10.3390/ph17050640)
Supplement: Supplementary file 1 [file pharmaceuticals-17-00640-s001.zip › pharmaceuticals-2968599-supplementary.pdf]

pharmaceuticals

Supplementary Material

Table S1. Predicted and observed values for pharmacokinetic parameters of hydromorphone. Predicted values are shown as the median of population simulations.

Study	Protocols	Methods	$C_{max}/C_{max\text{-}ss}$(ng/mL)	AUC/AUC_{ss}(ng*h/mL)	T_{max}(h)
Angst 2001	8 mg IR SD	Predicted	3.86	19.2	0.80
		Observed	4.74±1.76	18.0	0.80
		FE	0.81	1.07	1.00
	8 mg ER SD	Predicted	0.71	19.5	14.8
		Observed	0.77±0.33	22.9	12.0
		FE	0.92	0.85	1.23
	16 mg ER SD	Predicted	1.42	38.9	14.9
		Observed	1.45±0.43	43.6	15.0
		FE	0.98	0.89	0.99
	32 mg ER SD	Predicted	2.84	77.8	14.8
		Observed	2.41±0.85	81.0	16.5
		FE	1.18	0.96	0.90
Sathyan 2007	8 mg ER SD	Predicted	0.71	19.5	14.8
		Observed	0.93±1.01	19.5	12.0
		FE	0.76	1.00	1.23
	16 mg ER SD	Predicted	1.42	38.9	14.9
		Observed	1.69±0.78	40.8	16.0
		FE	0.84	0.95	0.93
	32 mg ER SD	Predicted	2.84	77.8	14.8
		Observed	3.25±1.37	80.3	16.0
		FE	0.87	0.97	0.93
	64 mg ER SD	Predicted	8.02	231	15.4
		Observed	6.61±1.75	179	16.0
		FE	1.21	1.29	0.96
Sathyan 2007 fasting	16 mg ER SD	Predicted	1.42	38.9	14.9
		Observed	1.11±0.21	38.8	16.0
		FE	1.28	1.00	0.93
Sathyan 2008	16 mg ER SD	Predicted	1.42	38.9	14.9
		Observed	1.37±0.32	40.6	16.0
		FE	1.04	0.96	0.93
Turgeon 2010	16 mg ER QD	Predicted	2.09	37.3	85.1
		Observed	2.32	/	84.0
		FE	0.90	/	1.01
	4 mg IR q6h	Predicted	2.95	9.44	90.8
		Observed	2.35	/	79.0
		FE	1.25	/	1.15

		GMFE	1.17	1.08	1.09

IR: Immediate release; ER: Extended release; SD: Singe dose; FE: Fold error; GMFE: Geometric mean fold error.

Table S2. Predicted and observed values for pharmacokinetic parameters of hydrocodone. Predicted values are shown as the median of population simulations.

Study	Protocols	Methods	$C_{max}/C_{max\text{-}ss}$(ng/mL)		AUC/AUC_{ss}(ng*h/mL)		T_{max}(h)	
			HYD	HYM	HYD	HYM	HYD	HYM
Hao 2011	5 mg uncoated SD	Predicted	9.76	0.10	63.1	1.09	0.80	1.20
		Observed	14.5	0.15	91.9	1.46	0.83	0.79
		FE	0.67	0.67	0.69	0.75	0.96	1.51
Harris 2016	60 mg uncoated SD	Predicted	117	/	762	/	0.80	/
		Observed	106	/	918	/	1.60	/
		FE	1.10	/	0.83	/	0.50	/
Darwish 2016	15 mg ER SD	Predicted	10.3	/	193	/	8.40	/
		Observed	10.1	/	155	/	6.00	/
		FE	1.02		1.24		1.40	/
	45 mg ER SD	Predicted	31.0	/	580	/	8.40	/
		Observed	28.6	/	565	/	8.00	/
		FE	1.08	/	1.03	/	1.05	/
Darwish 2015	15 mg ER SD	Predicted	10.3	/	193	/	8.40	/
		Observed	12.6	/	199	/	7.00	/
		FE	0.82	/	0.97	/	1.20	
	30 mg ER SD	Predicted	20.7	/	387	/	8.40	/
		Observed	20.7	/	382	/	8.00	/
		FE	1.00	/	1.01	/	1.05	/
	45 mg ER SD	Predicted	31.0	/	580	/	8.40	/
		Observed	30.3	/	592	/	8.00	/
		FE	1.02	/	0.98	/	1.05	/
	60 mg ER SD	Predicted	41.1	/	772	/	8.40	/
		Observed	41.2	/	766	/	8.00	/
		FE	1.00	/	1.01	/	1.05	/
	90 mg ER SD	Predicted	62.0	/	1159	/	8.40	/
		Observed	62.5	/	1189	/	8.00	/
		FE	0.99	/	0.97	/	1.05	/
Farr 2015	20 mg ER SD	Predicted	13.8	/	258	/	8.40	/
		Observed	22.7	/	345	/	8.00	/
		FE	0.60	/	0.75	/	1.05	/
	50 mg ER SD	Predicted	34.5	/	645	/	8.40	/
		Observed	46.3	/	846	/	6.16	/
		FE	0.75	/	0.76	/	1.36	/
Kapil 2015	20 mg ER SD	Predicted	11.3	0.15	258	4.37	14.6	15.6

	EM	Observed	15.9	0.19	328	3.80	18.0	16.1
		FE	0.71	0.79	0.79	1.15	0.81	0.97
	20 mg ER SD PM	Predicted	11.6	0.02	269	0.75	14.60	15.8
		Observed	16.8	0.06	347	0.64	18.0	18.0
		FE	0.69	0.33	0.78	1.17	0.81	0.88
Darwish 2014	45 mg ER SD	Predicted	31.0	/	580	/	8.40	/
		Observed	29.0	/	568	/	8.50	/
		FE	1.07	/	1.02	/	0.99	/
	45 mg ER BID	Predicted	53.4	/	580	/	8.60	/
		Observed	63.8	/	663	/	4.70	/
		FE	0.84	/	0.87	/	1.83	/
	90 mg ER SD	Predicted	62.0	/	1159	/	8.40	/
		Observed	56.4	/	1073	/	7.00	/
		FE	1.10	/	1.08	/	1.20	/
	90 mg ER BID	Predicted	106	/	1163	/	8.40	/
		Observed	123	/	1282	/	5.0	/
		FE	0.86	/	0.91	/	1.68	/
		GMFE	1.18	1.79	1.16	1.22	1.23	1.21

HYD: Hydrocodone; HYM: Hydromorphone; ER: Extended release; SD: Singe dose; FE: Fold error; GMFE: Geometric mean fold error.

Table S3. Clinical pharmacokinetic reports used in hydromorphone PBPK modeling.

Study	Dosage	Ethnicity	Population	Age, mean(range)	Number of subjects	Females proportion
Coda 1996	10 μg/kg iv	American	Healthy	27(21-38)	10	0
	20 μg/kg iv	American	Healthy	27(21-38)	10	0
	40 μg/kg iv	American	Healthy	27(21-38)	10	0
Angst 2001	8 mg IR SD	American	Healthy	27(21-34)	12	50%
	8 mg ER SD	American	Healthy	27(21-34)	12	50%
	16 mg ER SD	American	Healthy	27(21-34)	12	50%
	32 mg ER SD	American	Healthy	27(21-34)	12	50%
Sathyan 2007-1	8 mg ER SD	American	Healthy	33(20-50)	8	37.5%
	16 mg ER SD	American	Healthy	33(20-50)	8	37.5%
	32 mg ER SD	American	Healthy	33(20-50)	8	37.5%
	64 mg ER SD	American	Healthy	33(20-50)	8	37.5%
Sathyan 2007-2	16 mg ER SD fasting	American	Healthy	33.1(19-49)	30	30%
Sathyan 2008	16 mg ER SD	American	Healthy	21-45	24	NR
Turgeon 2010	16 mg ER QD	American	Healthy	NR	18	NR
	4 mg IR q6h	American	Healthy	NR	18	NR

iv: intravenous; IR: Immediate release; ER: Extended release; SD: Singe dose; NR: Not reported.

Table S4. Clinical pharmacokinetic reports used in hydrocodone PBPK modeling.

Study	Dosage	Ethnicity	Population	Age, mean(range)	Number of subjects	Females proportion
Hao 2011	5 mg uncoated SD	Chinese	Healthy	NR	12	NR
Harris 2016	60 mg uncoated SD	American	Healthy	38.9(21-54)	25	NR
Darwish 2016	15 mg ER SD	American	Healthy	49(41-59)	8	37%
	45 mg ER SD	American	Healthy	59.5(42-70)	14	43%
Darwish 2015	15 mg ER SD	American	Healthy	28.5(19-45)	60	34%
	30 mg ER SD	American	Healthy	28.5(19-45)	60	34%
	45 mg ER SD	American	Healthy	28.5(19-45)	60	34%
	60 mg ER SD	American	Healthy	28.5(19-45)	60	34%
	90 mg ER SD	American	Healthy	28.5(19-45)	60	34%
Farr 2015	20 mg ER SD	American	Healthy	22(19-33)	12	75%
	50 mg ER SD	American	Healthy	32(22-44)	30	7%
Kapil 2015	20 mg ER SD	American	Healthy	36(18-50)	24	50%
	20 mg ER SD+ Paroxetine	American	Healthy	36(18-50)	24	50%
Darwish 2014	45 mg ER SD	American	Healthy	26.5(20-45)	40	33%
	45 mg ER BID	American	Healthy	26.5(20-45)	40	33%
	90 mg ER SD	American	Healthy	28(20-44)	40	23%
	90 mg ER BID	American	Healthy	28(20-44)	40	23%

iv: intravenous; IR: Immediate release; ER: Extended release; SD: Singe dose; NR: Not reported.

Table S5. Input compound parameters for the fentanyl, alfentanil, and sufentanil PBPK models.

Parameters	Fentanyl	Alfentanil	Sufentanil
Lipophilicity	3.49	1.85	2.90
plasma fraction unbound	0.208	0.10	0.08
MW	336.48 g/mol	416.52 g/mol	386.60 g/mol
pKa	8.99	6.50	8.00
solubility	Various values provided in a table	0.992 mg/mL	8.0E-5 mg/mL
partition coefficients calculation	Diverse	Diverse	Diverse
cellular permeability	0.06 cm/min	6.88E-3 cm/min	6.78E-3 cm/min
Unspecific hepatic clearance	1.46 1/min	NA	NA
GFR fraction	1.0	0.06	1.0
$K_{m,CYP3A4}$	117 μmol/L, 82 μmol/L	NA	NA
$K_{cat,CYP3A4}$	20.6 1/min, 4.7 1/min	NA	NA
$K_{m,CYP3A7}$	596 μmol/L	NA	NA
$K_{cat,CYP3A7}$	5.22 1/min	NA	NA
$K_{m,P\text{-}gp}$	5.72 μmol/L	NA	NA
$K_{cat,P\text{-}gp}$	1.71 1/min	NA	NA
CYP3A4 specific clearance	NA	0.34 1/min	6.11 1/min

MW: molecular weight; pKa: acid dissociation constant; GFR: glomerular filtration; Km: Michaelis-Menten constant; kcat: V_{max} per recombinant enzyme.

The model files of the above compounds are publicly available at the Open Systems Pharmacology repository on Github (https://github.com/Open-Systems-Pharmacology?page=1).

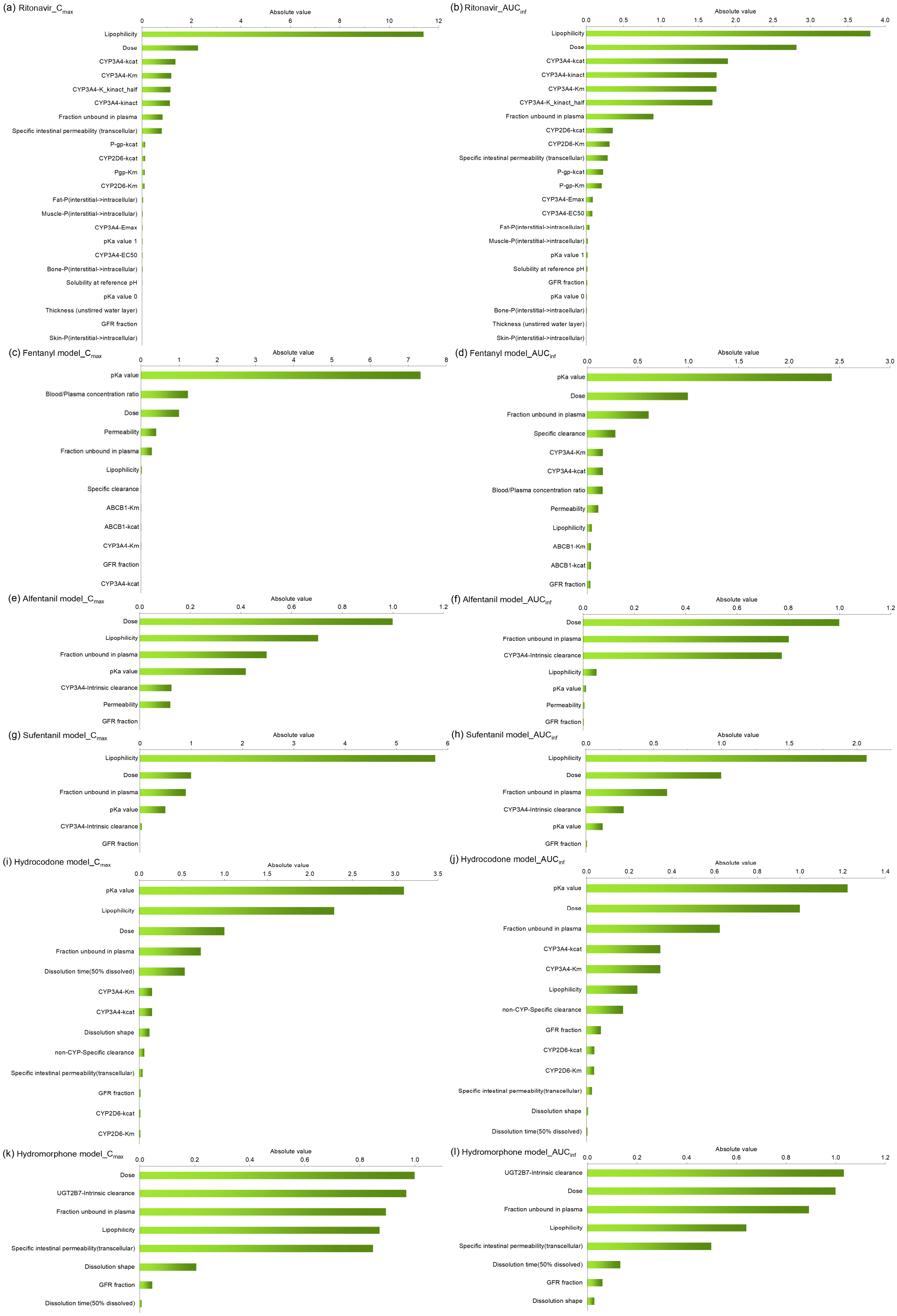
(a) Ritonavir_Cmax
Absolute value
0 2 4 6 8 10 12
Lipophilicity
Dose
CYP3A4-kcat
CYP3A4-Km
CYP3A4-K_kinact_half
CYP3A4-kinact
Fraction unbound in plasma
Specific intestinal permeability (transcellular)
P-gp-kcat
CYP2D6-kcat
Pgp-Km
CYP2D6-Km
Fat-P(interstitial->intracellular)
Muscle-P(interstitial->intracellular)
CYP3A4-Emax
pKa value 1
CYP3A4-EC50
Bone-P(interstitial->intracellular)
Solubility at reference pH
pKa value 0
Thickness (unstirred water layer)
GFR fraction
Skin-P(interstitial->intracellular)
(b) Ritonavir_AUCinf
Absolute value
0.0 0.5 1.0 1.5 2.0 2.5 3.0 3.5 4.0
Lipophilicity
Dose
CYP3A4-kcat
CYP3A4-kinact
CYP3A4-Km
CYP3A4-K_kinact_half
Fraction unbound in plasma
CYP2D6-kcat
CYP2D6-Km
Specific intestinal permeability (transcellular)
P-gp-kcat
P-gp-Km
CYP3A4-Emax
CYP3A4-EC50
Fat-P(interstitial->intracellular)
Muscle-P(interstitial->intracellular)
pKa value 1
Solubility at reference pH
GFR fraction
pKa value 0
Bone-P(interstitial->intracellular)
Thickness (unstirred water layer)
Skin-P(interstitial->intracellular)
(c) Fentanyl model_Cmax
Absolute value
0 1 2 3 4 5 6 7 8
pKa value
Blood/Plasma concentration ratio
Dose
Permeability
Fraction unbound in plasma
Lipophilicity
Specific clearance
ABCB1-Km
ABCB1-kcat
CYP3A4-Km
GFR fraction
CYP3A4-kcat
(d) Fentanyl model_AUCinf
Absolute value
0.0 0.5 1.0 1.5 2.0 2.5 3.0
pKa value
Dose
Fraction unbound in plasma
Specific clearance
CYP3A4-Km
CYP3A4-kcat
Blood/Plasma concentration ratio
Permeability
Lipophilicity
ABCB1-Km
ABCB1-kcat
GFR fraction
(e) Alfentanil model_Cmax
Absolute value
0.0 0.2 0.4 0.6 0.8 1.0 1.2
Dose
Lipophilicity
Fraction unbound in plasma
pKa value
CYP3A4-Intrinsic clearance
Permeability
GFR fraction
(f) Alfentanil model_AUCinf
Absolute value
0.0 0.2 0.4 0.6 0.8 1.0 1.2
Dose
Fraction unbound in plasma
CYP3A4-Intrinsic clearance
Lipophilicity
pKa value
Permeability
GFR fraction
(g) Sufentanil model_Cmax
Absolute value
0 1 2 3 4 5 6
Lipophilicity
Dose
Fraction unbound in plasma
pKa value
CYP3A4-Intrinsic clearance
GFR fraction
(h) Sufentanil model_AUCinf
Absolute value
0.0 0.5 1.0 1.5 2.0
Lipophilicity
Dose
Fraction unbound in plasma
CYP3A4-Intrinsic clearance
pKa value
GFR fraction
(i) Hydrocodone model_Cmax
Absolute value
0.0 0.5 1.0 1.5 2.0 2.5 3.0 3.5
pKa value
Lipophilicity
Dose
Fraction unbound in plasma
Dissolution time(50% dissolved)
CYP3A4-Km
CYP3A4-kcat
Dissolution shape
non-CYP-Specific clearance
Specific intestinal permeability(transcellular)
GFR fraction
CYP2D6-kcat
CYP2D6-Km
(j) Hydrocodone model_AUCinf
Absolute value
0.0 0.2 0.4 0.6 0.8 1.0 1.2 1.4
pKa value
Dose
Fraction unbound in plasma
CYP3A4-kcat
CYP3A4-Km
Lipophilicity
non-CYP-Specific clearance
GFR fraction
CYP2D6-kcat
CYP2D6-Km
Specific intestinal permeability(transcellular)
Dissolution shape
Dissolution time(50% dissolved)
(k) Hydromorphone model_Cmax
Absolute value
0.0 0.2 0.4 0.6 0.8 1.0
Dose
UGT2B7-Intrinsic clearance
Fraction unbound in plasma
Lipophilicity
Specific intestinal permeability(transcellular)
Dissolution shape
GFR fraction
Dissolution time(50% dissolved)
(l) Hydromorphone model_AUCinf
Absolute value
0.0 0.2 0.4 0.6 0.8 1.0 1.2
UGT2B7-Intrinsic clearance
Dose
Fraction unbound in plasma
Lipophilicity
Specific intestinal permeability(transcellular)
Dissolution time(50% dissolved)
GFR fraction
Dissolution shape

Figure S1. The sensitivity analysis for PBPK models used in this study. Sensitivity of the final model was measured as the relative change of a specific pharmacokinetic parameter after a single intravenous dose of fentanyl analogs or a single oral dose of ritonavir/hydrocodone/hydromorphone. A sensitivity value of +1.0 denotes that a 10% increase in the examined parameter causes a 10% increase in the pharmacokinetic parameter. The sensitivity values are presented as absolute values in figures.

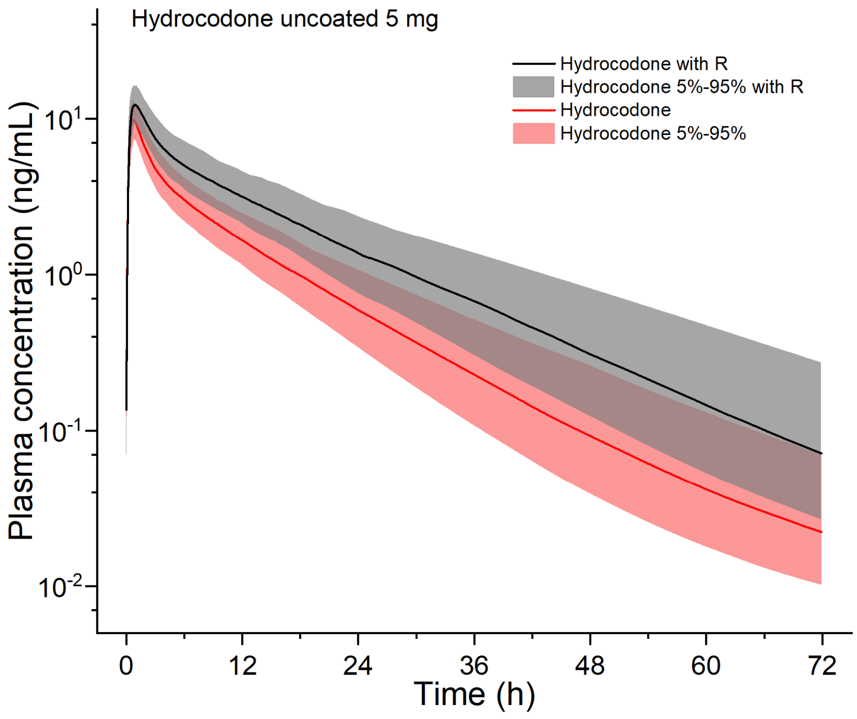

Figure S2. Simulated concentrations of a single-dose hydrocodone uncoated tablet in the presence of ritonavir.
